# Possible Race and Gender Divergence in Association of Genetic Variations with Plasma von Willebrand Factor: A Study of ARIC and 1000 Genome Cohorts

**DOI:** 10.1371/journal.pone.0084810

**Published:** 2014-01-17

**Authors:** Zhou Zhou, Fuli Yu, Ashley Buchanan, Yuanyuan Fu, Marco Campos, Kenneth K. Wu, Lloyd E. Chambless, Aaron R. Folsom, Eric Boerwinkle, Jing-fei Dong

**Affiliations:** 1 State Key Laboratory of Cardiovascular Disease, National Center for Cardiovascular Diseases, Fuwai Hospital, CAMS & PUMC, Beijing, China; 2 Human Genome Sequencing Center, Department of Human and Molecular Genetics, Baylor College of Medicine, Houston, Texas, United States of America; 3 Institute of Neurology, Tianjin Medical University General Hospital, Tianjin, China; 4 Department of Biostatistics, University of North Carolina, Chapel Hill, North Carolina, United States of America; 5 Section of Cardiology, Department of Medicine, Baylor College of Medicine, Houston, Texas, United States of America; 6 National Health Research Institutes, Taipei, Taiwan; 7 Division of Epidemiology & Community Health, School of Public Health, University of Minnesota, Minneapolis, Minnesota, United States of America; 8 Human Genetic Center, University of Texas School of Public Health, Houston, Texas, United States of America; 9 Puget Sound Blood Research Institute, Puget Sound Blood Center, Seattle, Washington, United States of America; 10 Division of Hematology, Department of Medicine, University of Washington School of Medicine, Seattle, Washington, United States of America; National Cerebral and Cardiovascular Center, Japan

## Abstract

The synthesis, secretion and clearance of von Willebrand factor (VWF) are regulated by genetic variations in coding and promoter regions of the *VWF* gene. We have previously identified 19 single nucleotide polymorphisms (SNPs), primarily in introns that are associated with VWF antigen levels in subjects of European descent. In this study, we conducted race by gender analyses to compare the association of VWF SNPs with VWF antigen among 10,434 healthy Americans of European (EA) or African (AA) descent from the Atherosclerosis Risk in Communities (ARIC) study. Among 75 SNPs analyzed, 13 and 10 SNPs were associated with VWF antigen levels in EA male and EA female subjects, respectively. However, only one SNP (RS1063857) was significantly associated with VWF antigen in AA females and none was in AA males. Haplotype analysis of the ARIC samples and studying racial diversities in the *VWF* gene from the 1000 genomes database suggest a greater degree of variations in the *VWF* gene in AA subjects as compared to EA subjects. Together, these data suggest potential race and gender divergence in regulating VWF expression by genetic variations.

## Introduction

von Willebrand factor (VWF) is a multimeric glycoprotein ligand essential in initiating hemostasis at sites of vessel injury [1]. It can also promote thrombosis by mediating platelet adhesion to activated endothelium and/or subendothelium at sites of ruptured atherosclerotic plaques, or aggregating platelets under pathological high shear stress found at sites of arterial stenosis [2]; [3]. An elevated plasma level of VWF is an independent risk factor for coronary heart disease (CHD), ischemic stroke, and peripheral artery disease [4]; [5], whereas low VWF antigen and activity could result in bleeding associated with von Willebrand disease (VWD) [6].

Plasma VWF antigen levels vary significantly among healthy individuals [7]; [8]. Both environmental and genetic factors contribute to this variation. The former includes conditions known to stimulate endothelial cells to secret VWF [9]; [10], whereas the latter plays a dominant role in determining a baseline level of circulating VWF [11]; [12]. ABO blood types greatly influence the baseline level of plasma VWF. Individuals with blood type O have a lower level of circulating VWF, resulting from an accelerated clearance of VWF from the circulation [13]. Genetic variations in the promoter and coding regions of the *VWF* gene are known to affect VWF levels [14]; [15]. We have previously identified intronic single nucleotide polymorphisms (SNPs) and their haplotypes that are associated with plasma VWF levels in 7,856 subjects of European descent (EA) from the Atherosclerosis Risk in Communities (ARIC) cohort [16]. We have also detected a significant ethnic diversity of variations in the *VWF* gene among subjects included in the 1000 genomes database [17]. In this study, we further examined potential race and gender differences in this association between VWF antigen and VWF gene variations. This study is important because AA subjects often have a higher level of circulating VWF antigen [7]; [8]; [18]. Recent studies by Bellissimo D. B., *et al* [19] and by us [17] show that some “mutations” that were originally associated with VWD in Caucasians are found in 10–20% of African subjects without evidence of clinical bleeding, even though some of these “VWD mutants” are dose-dependently associated with VWF levels in apparently healthy AA subjects [20]. Together, these data suggest that 1) the association between variations in the *VWF* gene and plasma levels of VWF antigen may vary by race and 2) this racial diversity may impact on how genetic variations influence VWF synthesis, secretion and clearance. In this study, we further examined the racial diversity in this association between VWF antigen and VWF gene variations between the EA and AA subjects from the ARIC cohort.

## Methods

The Atherosclerosis Risk in Communities (ARIC, http://www.cscc.unc.edu/aric/) is a prospective cohort study designed to assess subclinical atherosclerosis and clinical atherosclerotic events and cardiovascular risk factors [21]. Among 15,792 ARIC participants, 10,434 were included in this study after exclusion of subjects lacking data on 1) VWF antigen (n  =  280), 2) VWF SNPs (n  =  3,032), or 3) ABO genotypes (n  =  1,898). The study also excluded non-EA and non-AA individuals (n  =  48) and those who did not consent to DNA use (n  =  100). Covariates included ABO genotypes (O vs. non-O), age, gender, body mass index (BMI), hypertension, diabetes, and smoking status, all of which are known to affect VWF antigen levels. VWF measurements, SNP genotyping, haplotype construction, and data analyses were previously described [16]. A polyclonal VWF antibody was used to detect VWF antigen in plasma samples, but if this antibody binds VWF multimers from EA and AA subjects with an equal affinity has not been experimentally determined.

The difference between EA and AA subjects in allele frequencies of these VWF variants were further validated with information from the April 2012 Integrated Variant Set release of the 1000 Genomes Project (ftp://ftp.1000genomes.ebi.ac.uk/vol1/ftp/release/20110521/ALL.wgs.phase1_release_v3.20101123.snps_indels_sv.sites.vcf.gz). This database compiles information on genomic variations among 1,092 subjects from 14 ethnicities [25] and has been used to demonstrate a significant ethnic allelic diversity of VWF SNPs among Africans, Americans of European decent, Europeans and Asians [17].

## Results and Discussion

Characteristics of the 10,434 subjects are summarized in Table 1 and found to be consistent with our previous reports [16]; [22]. AA subjects (N  =  2,378) had significantly higher BMI and prevalences of diabetes and hypertension, but a lower prevalence of smoking compared to EA subjects (N  =  8,056). The mean level of plasma VWF varied significantly in race-by-gender groups. VWF levels found in female subjects of this ARIC cohort are comparable to a previous report on a different cohort by Millar C, *at al* [23]. ABO blood groups (defined as O type vs. non-O type) explained 12.6% of VWF variation when it was analyzed together with environmental covariates (gender, age, race, BMI, hypertension, diabetes and smoking status) in a linear regression model, slightly lower than 15.2% for EA subjects [16].

**Table 1:** Demographic and Plasma VWF for 10,434 ARIC Participants by Race.

Characteristics		Race		
		AA	EA	P value
Subject included	N (%)	2,378 (22.8)	8,056 (77.2)	
Gender	F	1,505	4,286	< 0.001
	M	873	3770	
BMI	Mean (s.d)	30.12 (6.37)	27.01 (4.67)	< 0.001
Diabetes	N (%)	452 (19.3)	726 (9.1)	< 0.001
Hypertension	N (%)	1,308 (55.0)	2,256 (27.8)	< 0.001
History of smoking*	N (%)	1,260 (53.1)	4,818 (59.8)	< 0.001
VWF [mean (s.d)]	F	128.15 (54.42)	108.44 (41.10)	< 0.001
	M	122.74 (51.16)	111.34 (42.36)	< 0.001

include current and former smokers.

We have previously identified 19 SNPs (17 intronic) in EA subjects that were associated with VWF antigen levels after adjustment for ABO and environmental covariates [16]. For that study, we were unable to study AA subjects because ABO genotype was imputed, a method that is not reliable for AA subjects. ABO has since been genotyped in the ARIC cohort, allowing us to analyze these 75 VWF SNPs for association with VWF antigen in EA and AA subjects, separately (Table S1) and to examine potential race and gender effects on the association. Fifteen SNPs (20%) were associated with overall VWF antigen levels after adjustments for environmental covariates and ABO (Table 2). The genotype frequency differed significantly between EA and AA subjects in 80% of these SNPs. Two of these SNPs (RS1063857 and RS12304995) had a reversed genotype frequency between EA and AA subjects. For the majority of these 15 positive SNPs, the minor alleles were associated with a higher level of VWF. Among these 15 positive SNPs, 13 and 10 reached statistical significance in EA males and EA females, respectively, with 5 (38.5%) and 2 (20%) of them being significant only in males and females, respectively.

**Table 2:** SNPs Associated with Plasma VWF Levels Analyzed for Race-by-Gender.

SNP	Race by Sex	Mean ± S.E. (Genotype frequency %)			P value^#^
		AA	AB	BB	
RS1063857 (Exon 18)	EA-F	104.8±0.9 (41%)	109.8±0.8 (46%)	115.3±1.6 (13%)	<.0001
	EA-M	106.5±1.0 (40%)	113.0±0.9 (47%)	119.7±1.7 (13%)	<.0001
	AA-F	117.7±3.2 (16%)	126.6±1.8 (50%)	136.0±2.2 (33%)	<.0001
	AA-M	113.5±4.2 (16%)	121±2.3 (50%)	128.3±2.7 (35%)	0.0076
RS216315 (Intron 22)	EA-F	91.4±5.7 (1%)	100.7±1.4 (16%)	110.1±0.6 (83%)	<.0001
	EA-M	97.9±6.4 (1%)	105.9±1.6 (15%)	112.374±0.7 (84%)	0.0001
	AA-F	123.6±5.1 (7%) *		128.7±1.3 (93%)	0.3382
	AA-M	117.9±5.9 (7%) *		122.9±1.7 (93%)	0.4186
RS11610629 (Intron 22)	EA-F	106.5±0.7 (56%)	110.3±0.9 (38%)	113.7±2.4 (6%)	0.0004
	EA-M	108.9±0.9 (55%)	113.3±1.0 (39%)	118.2±2.6 (6%)	<.0001
	AA-F	124.9±1.8 (54%)	131.1±2.1 (39%)	134.7±5.2 (7%)	0.0362
	AA-M	119.5±2.3 (49%)	127.5±2.5 (43%)	114.8±6.1 (8%)	0.0277
RS216318 (Intron 21)	EA-F	92.1±5.5 (1%)	100.7±1.4 (16%)	110.1±0.6 (83%)	<.0001
	EA-M	97.9±6.4 (1%)	105.8±1.6 (15%)	112.4±0.7 (84%)	<.0001
	AA-F	120.5±4.6 (8%) *		129.0±1.4 (92%)	0.0788
	AA-M	117.9±5.8 (8%) *		123.0±1.7 (92%)	0.4004
RS216295 (Intron 17)	EA-F	110.1±0.6 (82%)	101.6±1.4 (17%)	92.9±5.5 (1%)	<.0001
	EA-M	112.3±0.7 (83%)	106.4±1.6 (16%)	91.7±6.8 (1%)	<.0001
	AA-F	130.5±1.6 (66%)	125.3±2.4 (31%)	110.8±7.8 (3%)	0.0153
	AA-M	123.5±1.9 (68%)	120.2±3.1 (29%)	124.9±8.9 (3%)	0.6384
RS216298 (Intron 16)	EA-F	94.8±5.4 (1%)	101.2±1.4 (17%)	110.1±0.6 (82%)	<.0001
	EA-M	95.1±6.2 (1%)	106.0±1.6 (16%)	112. 5±0.7 (83%)	<.0001
	AA-F	113.0±8.1 (3%)	124.9±2.5 (26%)	130.1±1.5 (71%)	0.0351
	AA-M	120.2±9.2 (3%)	120.7±3.3 (25%)	123.3±1.9 (72%)	0.7643
RS216299 (Intron 16)	EA-F	96.3±5.0 (1%)	101.5±1.4 (17%)	110.0±0.6 (82%)	<.0001
	EA-M	95.5±5.9 (1%)	106.2±1.6 (16%)	112.4±0.7 (83%)	<.0001
	AA-F	112.0±7.9 (3%)	125.0±2.5 (26%)	130.2±1.5 (71%)	0.0242
	AA-M	121.1±8.8 (3%)	120.4±3.3 (25%)	123.3±1.9 (72%)	0.7458
RS12304995 (Intron 15)	EA-F	105.5±0.9 (39%)	109.5±0.8 (48%)	113.3±1.6 (13%)	<.0001
	EA-M	108.2±1.0 (39%)	112.3±0.9 (48%)	116.6±1.8 (13%)	<.0001
	AA-F	126.1±3.0 (18%)	127.0±1.8 (51%)	132.5±2.4 (30%)	0.1279
	AA-M	113.7±3.9 (17%)	126.2±2.3 (49%)	122.0±2.8 (34%)	0.0229
RS11609815 (Intron 24)	EA-F	106.7±0.7 (56%)	110.4±0.9 (38%)	112.0±2.2 (7%)	0.0015
	EA-M	108.9±0.9 (55%)	113.4±1.0 (38%)	118.5±2.4 (7%)	<.0001
	AA-F	126.3±2.2 (36%)	129.7±1.9 (48%)	128.4±3.2 (16%)	0.4996
	AA-M	121.8±2.8 (32%)	123.0±2.3 (50%)	122.8±3.9 (18%)	0.9463
RS11063995 (Intron 22)	EA-F	111.9±2.2 (6%)	110.4±0.9 (38%)	106.7±0.7 (56%)	0.0016
	EA-M	118.8±2.5 (7%)	113.2±1.0 (38%)	108.9±0.9 (55%)	<.0001
	AA-F	128.3±3.3 (16%)	130.3±1.9 (48%)	126.5±2.2 (36%)	0.2735
	AA-M	121.6±4 (17%)	123.6±2.3 (51%)	121.3±2.8 (31%)	0.7942
RS11612401 (Intron 22)	EA-F	112.0±2.2 (6%)	110.4±0.9 (38%)	106.7±0.7 (56%)	0.0017
	EA-M	118.6±2.4 (7%)	113.3±1.0 (38%)	108.9±0.9 (55%)	<.0001
	AA-F	137.2±10.5 (2%)	134.2±2.6 (24%)	126.2±1.5 (74%)	0.0232
	AA-M	112.3±10.1 (3%)	124.8±3 (29%)	122±1.9 (69%)	0.4386
RS1800380 (Exon 22)	EA-F	106.7±0.7 (56%)	110.3±0.9 (38%)	112.2±2.2 (6%)	0.0018
	EA-M	109.0±0.9 (55%)	113.2±1.0 (38%)	118.5±2.4 (7%)	<.0001
	AA-F	124.8±1.8 (50%)	131.7±2 (41%)	131.4±4.5 (9%)	0.0294
	AA-M	119.2±2.4 (45%)	127.11±2.4 (44%)	117.5±5.1 (11%)	0.0389
RS11609728 (Intron 21)	EA-F	112.3±2.2 (6%)	110.4±0.9 (38%)	106.7±0.7 (56%)	0.0016
	EA-M	118.5±2.4 (7%)	113.4±1.0 (38%)	108.9±0.9(56%)	<.0001
	AA-F	129.6±3 (20%) *		128.0±1.4 (80%)	0.6181
	AA-M	122.8±3.3 (24%) *		122.5±1.8 (76%)	0.9303
RS2239161 (Intron 15)	EA-F	109.8±0.6 (79%)	103.6±1.3 (19%)	96.4±4.5 (2%)	<.0001
	EA-M	112.4±0.7 (79%)	108.0±1.4 (20%)	100.5±5.2 (1%)	0.0028
	AA-F	128.8±1.4 (88%) *		124.3±3.9 (11%)	0.2801
	AA-M	123.5±1.7 (88%) *		112.0±4.6 (12%)	0.0207
RS2239160 (Intron 15)	EA-F	96.2±4.5 (2%)	103.3±1.3 (19%)	109.8±0.6 (79%)	<.0001
	EA-M	100.3±5.2 (1%)	107.5±1.4 (19%)	112.3±0.7 (79%)	0.0011
	AA-F	116.23±9.7 (2%)	129.2±2.7 (23%)	128.3±1.5 (75%)	0.4343
	AA-M		121.1±3.1 (27%) *	123.1±1.9 (73%)	0.5746

# comparing mean VWF antigen by genotype* Combined with heterozygote due to limited number of subjects.

In contrast, only one of these 15 positive SNPs (RS1063857) was associated with VWF levels in AA females and none in AA males (Table 2). RS1063857, which was associated with EA males and females, is a synonymous SNP located in exon 18 of the *VWF* gene. Nucleotide sequence in exon 18 encodes for the D’ domain that is involved in VWF multimerization [24] and binding FVIII [25]. This SNP also generated the strongest association in our previous study of EA subjects [16] and was associated with VWF antigen levels in a GWA study by the Cohorts for Heart and Aging Research in Genomic Epidemiology (CHARGE) consortium [26]. Together, these data suggest potential race and gender diversities in allele frequencies for these SNPs.

These race and gender diversities were further validated using information provided in the 1000 Genomes Project. Among the 15 positive SNPs, 7 (53.3%) had allele frequencies of 1.9 – 9.6 fold higher in Africans than in EA subjects and 1 (6.7%) was 2.7 fold more common in EA subjects (Table 3). As expected, Americans of European decent and European subjects had a highly comparable level of allele frequencies for all 15 VWF SNPs. However, 6 SNPs that differ most between Africans and EA were very rare in Asians (> 100 fold less frequent).

**Table 3:** Allele Frequency of VWF SNP in Four Ethnic Groups (%).

SNP	Ref. allele	Alt. allele	African	EA	European	Asian
RS1063857	A	G	65	27	38	8
RS216315	A	G	98	94	91	79
RS11610629	A	C	27	22	25	0.17
RS216318	A	C	98	93	91	79
RS216295	T	C	85	9	91	77
RS216298	C	T	87	91	91	77
RS216299	A	G	87	91	91	77
RS12304995	C	T	62	31	37	18
RS11609815	C	G	41	22	25	0.17
RS11063995	T	C	41	22	25	0.17
RS11612401	G	C	11	2	25	0.17
RS1800380	C	T	27	22	25	0.17
RS11609728	C	A	7	19	25	0.17
RS2239161	G	A	97	91	88	77
RS2239160	A	G	86	9	88	77

**Figure 1:**
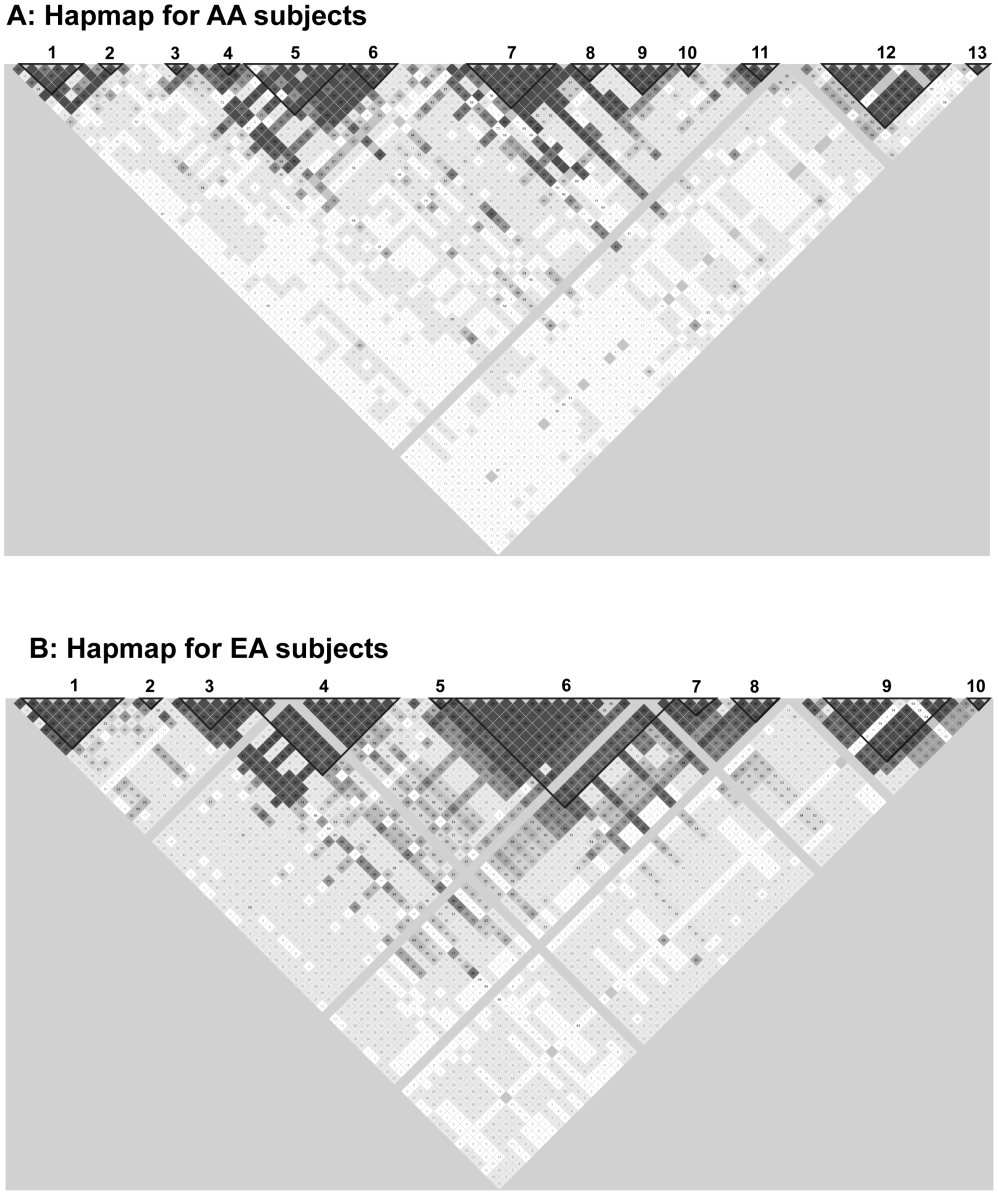
VWF haplotypes. The haplotypes were constructed using the default setting that (1) ignores pairview comparisons of markers more than 500 kb apart, (2) excludes subjects with more than 50% of missing genotypes, (3) examines haplotypes found 1% or more in the population, (4) removes markers with Hardy-Weinberg disequilibrium, and (5) uses R^2^ to define haplotype blocks.

In addition to individual SNPs, we also constructed haplotypes that included rare VWF SNPs in order to identify SNPs that are co-transmitted together. Using the fastPHASE 1.2 program, we identified 10 and 13 major haplotype blocks that are in linkage disequilibrium for EA and AA subjects, respectively (Figure 1). SNPs in each LD haplotype varied significantly between EA and AA subjects, indicating a highly diverse SNP co-aggregation between EA and AA subjects.

In conclusion, we have identified VWF SNPs that appear to be differentially associated with plasma levels of VWF antigen between EA and AA subjects as well as between male and female subjects. We did not detect a significant race-by-SNPs interaction for several reasons. First, the numbers of AA subjects were relatively small (N  =  2,378) compared to EA subjects (N  =  8,056) because the difference in VWF antigen associated with these intronic variations are expected to be small. However, this small sample size is partially compensated by higher allele frequencies in AA subjects for most of these SNPs (Table 2 & Table 3). Second, the *VWF* gene is more variable in AA subjects as suggested by their haplotypes (Figure 1). Finally, these *VWF* genetic variations may exert a smaller influence on AA subjects because of their relative higher baseline levels of VWF antigen. Covariates known to affect VWF antigen levels (ABO, age, gender, body mass index, hypertension, diabetes, and smoking status) were adjusted for the data analyses, but other covariates may exist and are not accounted for. These data need to be validated in larger cohorts of mix ethnicities, but they, nevertheless, suggest that the association between genetic and antigenic variations of VWF could be influenced by race and gender. This notion of racial divergence is consistent with recent reports that selective “mutations” previously associated with the bleeding disorder VWD were detected at 10–21% of AA subjects without documented bleeding [17]; [19]. The finding suggests that these “mutations” are unlikely to cause the disease. Our data, therefore, suggest caution in associating a specific SNP with a bleeding or thrombotic state without considering the impact of race and gender.

## Supporting Information

Table S1 **Genotype frequencies of VWF SNPs by race and gender.This table lists frequencies of all VWF SNPs that were studied and categorized based on race and gender.**
(DOCX)
