# Supplementary material for: Possible Race and Gender Divergence in Association of Genetic Variations with Plasma von Willebrand Factor: A Study of ARIC and 1000 Genome Cohorts
Source: PLoS One. 2014 Jan 17;9(1):e84810. doi: 10.1371/journal.pone.0084810 (PMC3894939; doi:10.1371/journal.pone.0084810)
Supplement: Table S1 — Genotype frequencies of VWF SNPs by race and gender.This table lists frequencies of all VWF SNPs that were studied and categorized based on race and gender. (DOCX) [file pone.0084810.s001.docx]

**Supplemental Tables**

**Table S1: Genotype frequencies of VWF SNPs by race and gender.**

|  | | | | **EA Females**  **(N=4,282)** | | | **EA Males**  **(N=3,774)** | | | **AA Females**  **(N=1,505)** | | | **AA Males**  **(N=873)** | | |  |
| --- | --- | --- | --- | --- | --- | --- | --- | --- | --- | --- | --- | --- | --- | --- | --- | --- |
|  | **SNP** | **A** | **B** | **AA** | **AB** | **BB** | **AA** | **AB** | **BB** | **AA** | **AB** | **BB** | **AA** | **AB** | **BB** | **Location** |
| 1 | rs933408 | T | G | 64 | 32 | 3 | 65 | 31 | 4 | 74 | 24 | 2 | 72 | 26 | 2 | Intron 51 |
| 2 | rs2270151 | G | A | 73 | 25 | 2 | 72 | 26 | 3 | 86 | 14 | 1 | 86 | 13 | 1 | Intron 50 |
| 3 | rs2270152 | T | G | 63 | 33 | 4 | 62 | 33 | 5 | 82 | 17 | 1 | 82 | 18 | 1 | Intron 49 |
| 4 | rs3759320 | T | C | 37 | 47 | 16 | 35 | 50 | 15 | 61 | 34 | 5 | 59 | 36 | 4 | Intron 47 |
| 5 | rs3759321 | G | A | 27 | 49 | 24 | 26 | 50 | 23 | 21 | 49 | 30 | 22 | 50 | 28 | Intron 47 |
| 6 | rs7958883 | A | T | 39 | 46 | 15 | 37 | 49 | 14 | 84 | 16 | 1 | 84 | 16 | 0 | Intron 47 |
| 7 | rs12317523 | C | T | 50 | 41 | 9 | 52 | 40 | 8 | 32 | 50 | 18 | 33 | 51 | 15 | Intron 47 |
| 8 | rs723190 | T | C | 89 | 11 | 0 | 89 | 11 | 0 | 77 | 22 | 1 | 76 | 22 | 2 | Intron 47 |
| 9 | rs11063953 | A | G | 52 | 40 | 8 | 54 | 38 | 8 | 53 | 39 | 8 | 54 | 40 | 6 | Intron 47 |
| 10 | rs12369177 | G | T | 38 | 47 | 15 | 38 | 48 | 15 | 57 | 38 | 5 | 58 | 37 | 5 | Intron 47 |
| 11 | rs4764478 | T | A | 60 | 34 | 6 | 61 | 34 | 5 | 61 | 33 | 6 | 62 | 32 | 6 | Intron 46 |
| 12 | rs7301070 | A | G | 43 | 46 | 11 | 42 | 46 | 12 | 54 | 38 | 8 | 49 | 42 | 9 | Intron 44 |
| 13 | rs216852 | C | G | 44 | 45 | 11 | 44 | 45 | 11 | 14 | 46 | 40 | 15 | 44 | 40 | Intron 43 |
| 14 | rs4764521 | G | A | 63 | 32 | 5 | 65 | 31 | 4 | 89 | 10 | 1 | 86 | 13 | 1 | Intron 43 |
| 15 | rs3819540 | G | A | 99 | 1 | 0 | 99 | 1 | 0 | 92 | 8 | 0 | 92 | 7 | 0 | Intron 42 |
| 16 | rs216855 | G | A | 54 | 39 | 7 | 55 | 38 | 7 | 26 | 50 | 24 | 25 | 50 | 25 | Intron 42 |
| 17 | rs216856 | C | T | 43 | 45 | 12 | 43 | 45 | 12 | 20 | 50 | 30 | 21 | 49 | 30 | Intron 42 |
| 18 | rs216865 | C | T | 43 | 45 | 12 | 43 | 45 | 12 | 21 | 50 | 29 | 22 | 50 | 28 | Intron 42 |
| 19 | rs6489686 | C | T | 72 | 26 | 2 | 72 | 25 | 2 | 64 | 32 | 4 | 63 | 34 | 3 | Intron 42 |
| 20 | rs11063975 | C | A | 84 | 15 | 1 | 85 | 15 | 1 | 93 | 7 | 0 | 97 | 3 | 0 | Intron 38 |
| 21 | rs216887 | C | T | 29 | 50 | 21 | 28 | 51 | 22 | 19 | 52 | 29 | 21 | 49 | 30 | Intron 38 |
| 22 | rs7968035 | G | A | 100 | 0 | 0 | 100 | 0 | 0 | 90 | 10 | 0 | 90 | 9 | 0 | Intron 38 |
| 23 | rs216888 | G | A | 29 | 50 | 21 | 28 | 51 | 22 | 19 | 52 | 29 | 21 | 50 | 29 | Intron 38 |
| 24 | rs10849375 | G | C | 85 | 14 | 1 | 84 | 15 | 1 | 94 | 5 | 0 | 97 | 3 | 0 | Intron 38 |
| 25 | rs216896 | T | C | 28 | 50 | 21 | 27 | 51 | 22 | 18 | 50 | 32 | 20 | 49 | 31 | Intron 38 |
| 26 | rs12579603 | A | G | 42 | 46 | 12 | 40 | 47 | 12 | 86 | 14 | 0 | 87 | 13 | 0 | Intron 37 |
| 27 | rs216903 | C | T | 29 | 50 | 21 | 28 | 51 | 22 | 32 | 50 | 18 | 32 | 51 | 17 | Intron 34 |
| 28 | rs216904 | T | C | 38 | 48 | 15 | 38 | 47 | 14 | 50 | 41 | 9 | 48 | 42 | 10 | Intron 34 |
| 29 | rs216905 | C | T | 58 | 36 | 6 | 59 | 36 | 5 | 74 | 24 | 2 | 71 | 26 | 3 | Intron 34 |
| 30 | rs216801 | G | A | 37 | 48 | 15 | 38 | 47 | 15 | 50 | 42 | 9 | 47 | 43 | 10 | Intron 34 |
| 31 | rs542993 | T | C | 27 | 50 | 23 | 27 | 50 | 23 | 51 | 41 | 8 | 51 | 41 | 8 | Intron 34 |
| 32 | rs216308 | C | T | 35 | 47 | 17 | 35 | 47 | 18 | 77 | 20 | 2 | 78 | 19 | 3 | Intron 31 |
| 33 | rs1800385 | C | A | 83 | 16 | 1 | 83 | 16 | 1 | 95 | 5 | 0 | 96 | 4 | 0 | Exon 28 |
| 34 | rs216312 | C | T | 30 | 50 | 20 | 29 | 52 | 19 | 57 | 39 | 4 | 55 | 39 | 6 | Intron 27 |
| 35 | rs216313 | A | G | 88 | 12 | 1 | 88 | 12 | 0 | 97 | 3 | 0 | 98 | 2 | 0 | Intron 27 |
| 36 | rs2854871 | T | C | 85 | 15 | 1 | 85 | 15 | 1 | 77 | 22 | 1 | 80 | 19 | 1 | Intron 24 |
| 37 | rs11609815 | C | G | 56 | 38 | 7 | 55 | 38 | 7 | 36 | 49 | 16 | 32 | 50 | 18 | Intron 24 |
| 38 | rs11063995 | T | C | 56 | 38 | 6 | 55 | 38 | 7 | 36 | 49 | 16 | 32 | 51 | 17 | Intron 22 |
| 39 | rs216315 | G | A | 83 | 16 | 1 | 84 | 15 | 1 | 94 | 6 | 0 | 93 | 7 | 0 | Intron 22 |
| 40 | rs11612401 | G | C | 56 | 38 | 6 | 55 | 38 | 7 | 74 | 24 | 2 | 69 | 29 | 3 | Intron 22 |
| 41 | rs11610629 | A | C | 56 | 38 | 6 | 55 | 39 | 6 | 54 | 40 | 6 | 50 | 43 | 7 | Intron 22 |
| 42 | rs1800380 | C | T | 56 | 38 | 6 | 55 | 38 | 7 | 50 | 42 | 8 | 46 | 44 | 10 | Exon 22 |
| 43 | rs216318 | C | A | 83 | 16 | 1 | 84 | 15 | 1 | 92 | 8 | 0 | 92 | 8 | 0 | Intron 21 |
| 44 | rs11609728 | C | A | 56 | 38 | 6 | 56 | 38 | 7 | 81 | 19 | 1 | 77 | 22 | 1 | Intron 21 |
| 45 | rs216327 | C | T | 43 | 44 | 12 | 43 | 45 | 12 | 64 | 33 | 3 | 60 | 34 | 6 | Intron 18 |
| 46 | rs216329 | C | T | 43 | 44 | 12 | 43 | 45 | 12 | 64 | 33 | 3 | 60 | 34 | 6 | Intron 18 |
| 47 | rs1063857 | A | G | 41 | 46 | 13 | 40 | 47 | 13 | 16 | 50 | 33 | 15 | 48 | 37 | Exon 18 |
| 48 | rs216295 | C | T | 82 | 17 | 1 | 83 | 16 | 1 | 66 | 31 | 3 | 69 | 27 | 3 | Intron 17 |
| 49 | rs16932374 | C | T | 100 | 0 | 0 | 100 | 0 | 0 | 68 | 29 | 3 | 68 | 29 | 3 | Exon17 |
| 50 | rs216298 | T | C | 82 | 17 | 1 | 83 | 16 | 1 | 71 | 27 | 3 | 73 | 24 | 3 | Intron 16 |
| 51 | rs216299 | G | A | 82 | 17 | 1 | 83 | 16 | 1 | 71 | 27 | 3 | 73 | 24 | 3 | Intron 16 |
| 52 | rs2239161 | A | G | 79 | 20 | 2 | 79 | 20 | 1 | 89 | 11 | 0 | 88 | 12 | 0 | Intron 15 |
| 53 | rs2239160 | G | A | 79 | 19 | 2 | 79 | 19 | 1 | 75 | 23 | 2 | 73 | 25 | 2 | Intron 15 |
| 54 | rs7954351 | A | T | 52 | 41 | 7 | 52 | 40 | 8 | 20 | 49 | 32 | 18 | 50 | 31 | Intron 15 |
| 55 | rs12304995 | C | T | 40 | 48 | 13 | 39 | 48 | 13 | 19 | 52 | 30 | 17 | 49 | 34 | Intron 15 |
| 56 | rs10744696 | C | A | 36 | 48 | 16 | 35 | 48 | 16 | 54 | 39 | 7 | 50 | 44 | 7 | Intron 13 |
| 57 | rs7139057 | T | G | 67 | 30 | 4 | 67 | 30 | 4 | 76 | 23 | 2 | 72 | 27 | 1 | Intron 13 |
| 58 | rs12319392 | G | T | 84 | 15 | 1 | 84 | 15 | 1 | 44 | 45 | 10 | 44 | 45 | 11 | Intron 13 |
| 59 | rs2109122 | T | C | 85 | 14 | 1 | 86 | 14 | 1 | 45 | 45 | 10 | 45 | 45 | 10 | Intron 13 |
| 60 | rs3213721 | C | T | 100 | 0 | 0 | 100 | 0 | 0 | 100 | 0 | 0 | 100 | 0 | 0 | Intron 8 |
| 61 | rs1800387 | A | T | 95 | 5 | 0 | 94 | 6 | 0 | 35 | 48 | 17 | 32 | 50 | 18 | Exon 8 |
| 62 | rs7135976 | T | C | 26 | 50 | 25 | 25 | 50 | 25 | 6 | 36 | 58 | 6 | 36 | 58 | Intron 6 |
| 63 | rs763580 | G | C | 51 | 41 | 8 | 53 | 40 | 8 | 86 | 14 | 1 | 85 | 15 | 0 | Intron 6 |
| 64 | rs2238103 | A | T | 41 | 46 | 13 | 42 | 45 | 13 | 56 | 38 | 6 | 55 | 38 | 7 | Intron 6 |
| 65 | rs1610056 | C | G | 75 | 24 | 2 | 74 | 24 | 2 | 64 | 33 | 4 | 63 | 34 | 3 | Intron 6 |
| 66 | rs2239147 | T | C | 75 | 24 | 2 | 74 | 24 | 2 | 64 | 33 | 3 | 63 | 34 | 3 | Intron 6 |
| 67 | rs2239145 | C | T | 75 | 24 | 2 | 74 | 24 | 2 | 64 | 33 | 4 | 63 | 34 | 3 | Intron 6 |
| 68 | rs2239144 | C | A | 74 | 24 | 2 | 74 | 24 | 2 | 63 | 33 | 4 | 63 | 34 | 3 | Intron 6 |
| 69 | rs2239143 | A | G | 100 | 0 | 0 | 99 | 1 | 0 | 99 | 1 | 0 | 99 | 1 | 0 | Intron 6 |
| 70 | rs11064019 | A | G | 75 | 24 | 2 | 74 | 24 | 2 | 65 | 31 | 4 | 63 | 33 | 4 | Intron 6 |
| 71 | rs11064020 | T | C | 75 | 24 | 2 | 74 | 24 | 2 | 65 | 32 | 3 | 64 | 33 | 3 | Intron 6 |
| 72 | rs11064021 | A | G | 75 | 24 | 2 | 74 | 24 | 2 | 64 | 33 | 4 | 63 | 34 | 3 | Intron 5 |
| 73 | rs41388848 | T | C | 93 | 7 | 0 | 93 | 7 | 0 | 58 | 36 | 6 | 58 | 37 | 5 | Intron 5 |
| 74 | rs12307072 | A | T | 42 | 45 | 13 | 40 | 47 | 13 | 45 | 44 | 11 | 47 | 43 | 10 | Intron 3 |
| 75 | rs10849385 | A | G | 40 | 46 | 14 | 38 | 49 | 14 | 9 | 42 | 50 | 10 | 40 | 50 | Intron 3 |
